# Reproducibility and Validity of a Stroke Effectiveness Test in Table Tennis Based on the Temporal Game Structure

**DOI:** 10.3389/fpsyg.2019.00427

**Published:** 2019-02-28

**Authors:** Taisa Belli, Milton Shoiti Misuta, Pedro Paulo Ribeiro de Moura, Thomas dos Santos Tavares, Renê Augusto Ribeiro, Yura Yuka Sato dos Santos, Karine Jacon Sarro, Larissa Rafaela Galatti

**Affiliations:** ^1^Interdisciplinary Research Group on Racket Sports (GRIPER), School of Applied Sciences, University of Campinas, Limeira, Brazil; ^2^Laboratory of Biomechanics and Instrumentation (LABIN), School of Applied Sciences, University of Campinas, Limeira, Brazil; ^3^Sport Pedagogy Laboratory (LEPE), School of Applied Sciences, University of Campinas, Limeira, Brazil; ^4^Laboratory of Instrumentation for Biomechanics (LIB), School of Physical Education, University of Campinas, Campinas, Brazil

**Keywords:** racket sports, sport-specific test, sport pedagogy, speed, accuracy, performance analysis

## Abstract

**Purpose:** This study aimed to develop a stroke effectiveness test in table tennis based on the temporal game structure to assess the ball speed and ball placement of the players, with a purpose to analyze its reproducibility and validity.

**Methods:** Nineteen male table tennis players participated in this study. The test was performed twice during the first session and once during the second session to assess the intrasession and intersession reproducibility, respectively. Moreover, the test was examined on its ability to discriminate between regional (*n* = 10) and local performance-level (*n* = 9) players and on the relationship between the test results and the table tennis performance to assess the discriminant and concurrent validity, respectively. In general, the test consisted of 11 simulated rallies of 2–5 balls with the effort and rest ratio of 0.5, and focused on attack with offensive strokes at defensive balls delivered by a robot randomly between the left and right positions on the table.

**Results:** Ball speed, ball placement, and ball speed-ball placement index showed satisfactory reliability (ICC range 0.78–0.96, *P* < 0.05) and agreement (CV range 2.7–16.2%) outcomes. Additionally, the Bland–Altman plots show the systematic error of the analyses closer to 0, and that most values were within the limits of agreements. Concerning validity analyses, regional players had higher scores of ball placement (+51.3%; *P* = 0.01, ES = 1.33) and ball speed-ball placement index (+56.1%; *P* = 0.0009, ES = 1.87) as well as made fewer errors (-25.4%; *P* = 0.017, ES = 1.20) than local players. Moreover, ball placement (*r* = -0.79, *P* = 0.04), ball speed-ball placement index (*r* = -0.78, *P* = 0.04), and percentage error (*r* = 0.88, *P* = 0.01) presented a strong and significant correlation with table tennis performance. However, ball speed was slightly different between the regional than local players (+1.7%; *P* = 0.78, ES = 0.13) and this variable was not related to table tennis performance (*r* = 0.32, *P* = 0.49).

**Conclusion:** Our findings show evidences that the test is reproducible. Moreover, discriminant and concurrent validity are confirmed for ball placement and ball speed-ball placement index.

## Introduction

Table tennis is an intermittent sport comprising periods of effort and rest (Lees, 2003). It is one of the fastest ball games in the world, where the player is required to use a body of competencies (e.g., technical, tactical, and physical skills) (Faber, 2016) within a finite time duration to play during each rally of the match (Sève et al., 2002; Suzuki and Yamamoto, 2015). In accordance with this, the match characteristics of table tennis should be used by coaches to plan teaching and training, aimed at achieving an optimal performance by the athletes (Sève et al., 2002; Zagatto et al., 2010; Kondric et al., 2013). Thus, it is relevant that the protocols used to test the table tennis players represent the table tennis match (Kondric et al., 2013). Herein, we address these issues and focus on the development of a newly technical test based on the temporal characteristics of the match.

Previous investigations have proposed several tests to evaluate the perceptual-motor skills (Faber et al., 2014a,b, 2015), aerobic and anaerobic parameters (Morel and Zagatto, 2008; Zagatto et al., 2008, 2011; Zagatto and Gobatto, 2012), and technical skills (Le Mansec et al., 2016) in table tennis players. Almost most of the tests for measuring physiological and technical skills were applied in a specific table tennis exercise (i.e., in an ecological task), their protocols were characterized by a continuous profile, with use of only forehand (FH) offensive strokes at balls sent by a robot at fixed areas of ball contact with the table. Zagatto et al. (Morel and Zagatto, 2008; Zagatto et al., 2008, 2011; Zagatto and Gobatto, 2012) developed a number of specific table tennis tests to assess physiological parameters, which in general comprised incremental (e.g., intensities incremented every 2–3 min – effort periods – interspersed with pauses of 30 s until voluntary exhaustion) and constant (e.g., 3-4 effort periods from 2 to 10 min, separated by a minimum break of 2 h) protocols. In these studies, balls were sent systematically at 2–4 fixed areas on the table. Moreover, Le Mansec et al. (2016) developed a technical test characterized by 45 continuous topspin strokes at balls sent every 3 s (2 min and 15 s of effort) in only one position to the center of the table.

However, table tennis is characterized by short rallies (3.4 s. on average) comprised few strokes (4.0 shots per rally on average), each stroke performed every 1.7 s. (35.3 shots per min on average), with longer pauses in between rallies (11.6 s. on average) (Zagatto et al., 2010; Leite et al., 2017). This sport includes a wide range of FH and backhand (BH) techniques performed at balls bounce in different playing surfaces over the table (Munivrana et al., 2015a,b). In addition, investigations show evidences the rally length is fairly consistent among players with different performance-level (3.2–3.6 s.) whereas rest period in between rallies is lower in regional players (7.0 s) than athletes at national (9.3 s) and elite (18.6 s) performance-level. It results in higher values of the effort and rest ratio (0.50) at regional in relation to national players (0.34) and considerably higher values than at international athletes (0.18) (Zagatto et al., 2010; Leite et al., 2017). A table tennis test based on all these aspects is relevant to evaluate players in match-like conditions, like we can see in other racket sports, as tennis (Vergauwen et al., 1998; Kolman et al., 2017). However, to our knowledge, no study purposes to develop a reliable and valid test that includes the aforementioned characteristics and evaluates stroke effectiveness in table tennis players.

Technical skills, mainly characterized by strokes, are the basis for the execution of adequate tactics in a given situation (Wang et al., 2013). Stroke may be evaluated based on the mechanical aspects of technique and how skills are performed (i.e., stroke analysis) as well as it may be assessed based on the outcomes of skills performed, irrespective of how correctly a skill has been performed (i.e., stroke effectiveness) (O’Donoghue et al., 2013). Ball velocity and placement have been used in tests applied to racket sports to examine stroke effectiveness (Vergauwen et al., 1998; Le Mansec et al., 2016; Kolman et al., 2017). Munivrana et al. (2015b) evidenced that the ball speed and ball placement are amongst the main basic tactical means for table tennis players realize their own tactical ideas during the match. These are recognized as important elements that the players must master to play the match successfully (Qun et al., 1992; Malagoli Lanzoni et al., 2011). Faster ball speeds may induce favorable conditions to win the rally, since it imposes lesser time for the opponent to react (Le Mansec et al., 2016). Moreover, players use the ball placement to avoid the opponent’s preferred strokes (Malagoli Lanzoni et al., 2014), exploit opponent’s weaknesses and to move the ball out of the opponents control. Ball speed and ball spin, that is often used during the strokes to increase the accuracy of ball placement, are interrelated and restricted each other (Qun et al., 1992). Indeed, the speed-accuracy trade-off hypothesis states that movements become less accurate the faster we performed them (Fitts, 1954). In accordance with this, a recent investigation calculated the ball speed–ball placement index and demonstrated that this index is relevant to discriminate the players with different levels of performance (Le Mansec et al., 2016).

On the basis of the aforementioned statement, this study aims to develop a stroke effectiveness test based on the temporal structure of table tennis matches that may be able to assess the abilities of the players to perform ball speed and ball placement, with a purpose to assess the reproducibility [i.e., the results of the test are consistent when the players perform the test repeatedly (Hopkins, 2000; De Vet et al., 2006)] and validity [i.e., the test measures what it purports to measure (Impellizzeri and Marcora, 2009)] of the test. Reproducibility and validity are the two most important attributes that warrant consideration in the evaluation of new instruments in sports science (Hopkins, 2000). We focus the development of the test applied to regional performance-level players in a tactical situation of offensive strokes performed at defensive balls.

## Materials and Methods

### Participants

In total 19 male table tennis players participated in this study. Ten players were classified as the regional performance-level group (RP) according criteria proposed by Zagatto et al. (2010), which includes athletes that had more than 5 years of systematic and regular training, and that competed in regional and national tournaments. Regional players were recruited from the Southeastern region, Brazil [i.e., the region detected as the one with better conditions for athlete’s development in Brazil (Tozetto et al., 2017)] via table tennis training centers, clubs, and coaches. Local-performance level group (LP) was composed of nine undergraduate students with low experience in table tennis and without participation in regional or national tournaments. Table 1 presents the characteristics of each group. Regional players had significantly more years of table tennis experience (*P* < 0.001) and trained more hours per week (*P* < 0.0001) than local players. This study was carried out in accordance with the recommendations of “Ethics Research Committee of the University of Campinas” with written informed consent from all subjects. All subjects gave written informed consent in accordance with the Declaration of Helsinki. The protocol was approved by the “Ethics Research Committee of the University of Campinas (UNICAMP)” (n. 1.928.165/2017).

**Table 1:** Demographic and racket grip for the regional (RP) and local (LP) performance-level groups.

	RP (*N* = 10)	LP (*N* = 9)	*P* Value
Age (years)	23.9 ± 1.8	24.3 ± 2.6	0.91
Height (cm)	176.9 ± 2.1	174.6 ± 3.3	0.54
Weight (kg)	79.8 ± 3.1	68.1 ± 5.7	0.08
Table Tennis Experience (years)	7.5 ± 0.9	2.2 ± 0.3	<0.001
Training volume (hours/week)	10.0 ± 0.9	3.2 ± 0.5	<0.001
Racket Grip (Penholder/Classical)	1/9	3/6	


*Data are expressed as mean ± SEM.*

### Research Design

First, the reproducibility was examined with 16 players performed a session of a sport-specific test (duration ∼18 min), which started with a training-phase of warm up to familiarize them with the procedure. Furthermore, each player had to undergo the testing-phase twice to assess the intrasession reproducibility, with an 8-min resting period in between. Of the 16 players initially evaluated, 12 participated in the second session to assess the intersession reproducibility, which also included the training and testing-phases. Both sessions were separated by time duration of 2–5 days. In the second part of the study, the validity of the test was evaluated. The test was examined on its ability to discriminate between regional (*n* = 10) and local performance- level (*n* = 9) players and on the relationship between the test results and the table tennis performance. Known-groups validity is one approach to examine the construct validity of a test when there is no “gold standard.” For this purpose, the test is administered to two groups that are known to have different levels of the construct to confirm whether the hypothesized difference is reflected in the scores of the two groups (Davidson, 2014). This approach has been used in validity studies applied to table tennis (Faber et al., 2014a,b). Concerning concurrent validity, table tennis performance was evaluated as the ranking determined by simulated tournament among the subjects.

### Development of a Stroke Effectiveness Test Based on the Temporal Game Structure of Table Tennis Matches

#### Training-Phase

The training-phase comprised three steps. In the first step, athletes played 1 min of balls sent every 3 s in only one position to the center of the table. In the second step, athletes played eight rallies, each one comprised 2–5 balls sent by a robot. Rallies were delivered in a random order. Balls were sent every 1.1 s in only one position to the center of the table. The effort and pause ratio between rallies of 0.5 was used within this step. Finally, in the third step, athletes simulated the testing-phase. During the entire training-phase, the ball was delivered by the robot (Robo-Pong 2050, Newgy, Hendersonville, TN, United States) at 25 km/h with backspin, placing it from 100 to 120 cm away from the net, and the players were instructed to play with FH or BH offensive strokes. Each step of the training-phase was performed twice, and with 2–3 min of resting period between each trial.

#### Testing-Phase

The test aimed to simulate 11 rallies of a table tennis match. Each rally comprised 2–5 balls delivered by the robot (i.e., 4–10 shots per rally) at 25 km/h and frequency of 54 balls ⋅ min^-1^ (i.e., each ball was delivered at every 1.1 s). Players performed four rallies of two balls lasting 2.2 s each (∼36% of the test), four rallies of three balls lasting 3.3 s each (∼36% of the test), two rallies of four balls lasting 4.4 s each (∼18% of the test) and one rally of five balls lasting 5.5 s each (∼9% of the test); in total, 33 balls were delivered during the test. The rallies occurred in a random but programmed order (4, 2, 3, 3, 2, 5, 2, 4, 2, 3, 3 balls delivered), and the players were unaware of this order. The effort and pause ratio between rallies of 0.5 was used within the test. Temporal characteristics of the test were based on those found in regional table tennis players during the official matches. These authors reported 1–12 shots per rally; 14–75 shots ⋅ min^-1^; 35.4% of the rallies lasting 1.5–2.5 s, 24.7% lasting 2.5–3.5 s, 15.6% lasting 3.5–4.5 s, 9% lasting 4.5–5.5 s; and the effort and rest ratio of 0.5. (Zagatto et al., 2010).

**FIGURE 1:**
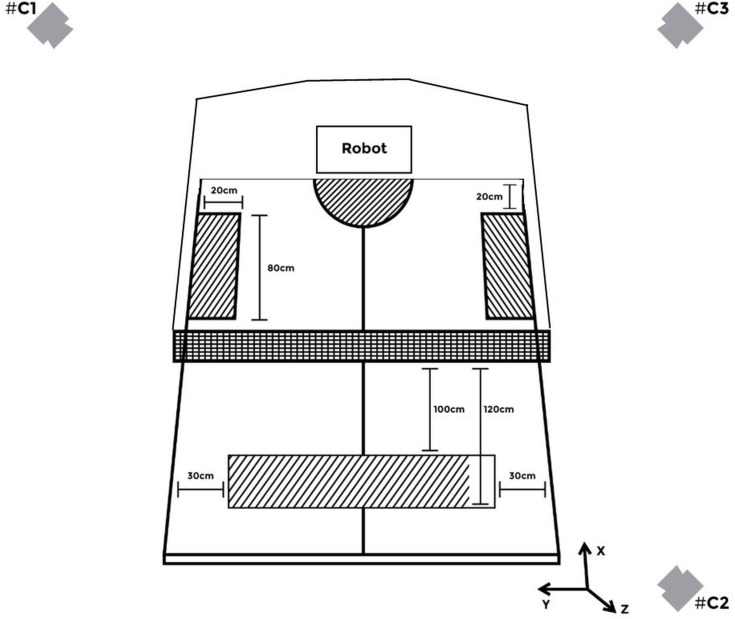
Schematic of the top view of the experimental setup. Shaded areas closer to the robot correspond to the three targets that the players could to reach, and the shaded area the other side of the net corresponds to the target zone the balls was thrown by the robot. #C1 = camera 1, #C2 = camera 2, #C3 = camera 3.

The ball was delivered by the robot with backspin, placing it from 100 to 120 cm away from the net and from 30 cm away from the sides (right and left). Balls were thrown by the robot randomly between the left and right positions within this target zone. Players could play with FH or BH offensive strokes. The strokes were chosen by the players during the test. The three targets were in strategic locations, in accordance with previously proposed by Le Mansec et al. (2016). Two rectangular targets (80 cm length, 20 cm width) were positioned on the sides (right and left side of the robot) of the table, 20 cm from the edge of the table. The third target was a semicircle of 25 cm in diameter positioned at the center of the table and close to its edge (Figure 1). Players could hit the three targets in a sequence that they decided, with the aim to realize their own tactical ideas to win the rally during the test, as they would during an official match. The performance assessment (i.e., the accuracy score and the performance index (PI), as described further) was explained to the players before starting the test. During all tests, a playing context similar to those observed during official matches was maintained.

### Data Collection and Processing

Three digital video cameras (Cassio EXFH25, 240 Hz) were positioned at 3 m height in order to cover the entire table and the movement of the athlete in the image view (Figure 1). The DVideo System (Campinas, Brazil) (Figueroa et al., 2008) was used for image calibration, ball tracking and 3D coordinate’s reconstruction. The images were calibrated using 196 reference points with known coordinates, placed around the testing volume. A tridimensional orthogonal reference system was defined considering the *x*-axis to be horizontally positive at front of the athlete, *y*-axis to be horizontally positive to the left and *z*-axis to be vertically positive upward. The positions of the reference points were used to generate the calibration parameters for the cameras using the direct linear transformation method (Abdel-Aziz and Karara, 1971).

The position of the ball was tracked manually by previously trained operators. The ball was tracked in each frame from contact with the racket to contact with the table. The calibration parameters and the position of the ball in the video sequences were used to reconstruct the tridimensional coordinates of the ball. For data smoothing, a second order polynomial was adjusted to the ball’s position using the MATLAB (2014R) environment. Moreover, the ball stroke velocity of successful shots (i.e., excluded all the shots that were hit out and into the net) was calculated right after the contact with the racket; thus, the average ball speed was calculated. The 3D position of each successful ball was projected on the table to identify the region of ball bounce on the table and to compute the accuracy score. The following procedure was used: when the ball reached the target, two points were granted; one point when the ball reached the table but did not touch the target and zero point when a fault was committed (Le Mansec et al., 2016). This procedure gave a score between 0 and 66 for each series. Finally, considering the ball speed–ball placement interrelation, a PI was calculated to link these two parameters with the following formula proposed by Le Mansec et al. (2016):

PI=average speed of the series × accuracy score/100

### Table Tennis Performance by Simulated Tournament

Table tennis performance was considered as the ranking estimated by means of a repechage tournament among the study players, which each athlete could lose one match and still be able to attain first place. The athlete was eliminated from the tournament after losing two matches. All table tennis matches were performed following the rules of the International Table Tennis Federation and played with a maximum of seven sets (the winner was required to win four-sets). The top three players were awarded trophies and medals.

### Statistical Analysis

Normal distribution and homogeneity of the data were verified by the Shapiro–Wilk and Levene’s tests, respectively. For the intra- and inter-operator reliability, the intraclass correlation (ICC) was used by evaluating the landing position of balls in one series by three operators (three times each operator). The reproducibility of the measurements was tested using intraclass correlation (ICC as two-way random model, absolute agreement), typical error (TE), smallest detectable difference (SDD), and coefficient of variation (CV) (Hopkins, 2000; De Vet et al., 2006). ICC of 0.70 or more was considered to be acceptable to prove the reliability of the test. TE, SDD, and CV were calculated as agreement parameters. Additionally, Bland–Altman analyses were also applied to provide a visual representation of measurement errors against true values (Bland and Altman, 1986). Discriminant validity was evaluated by comparing the scores on the ball speed, accuracy score, PI, and percentage error of the regional and local performance-level players using Student’s *t*-test for independent groups. Effect size (ES) was also calculated to determine the magnitude of the difference between comparisons. The threshold adopted was: trivial (0–0.19), small (0.20–0.49), medium (0.50–0.79), and large (0.80 and greater) (Cohen, 1992). The concurrent validity was investigated by correlating the ball speed, accuracy score, PI, and percentage error with the table tennis performance using the Spearman’s correlation coefficient. Statistical significance was set at *P* < 0.05. Statistical procedures were carried out using IBM SPSS Statistics 24 for Windows (IBM Corp., Armonk, NY, United States) and GPOWER 3.1 software.

## Results

**FIGURE 2:**
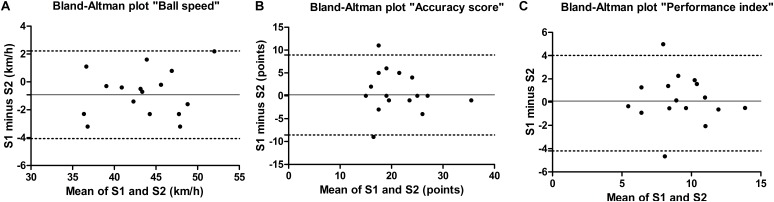
Bland–Altman plots for **(A)** ball speed, **(B)** accuracy score, and **(C)** performance index. The continuous line represents the mean difference between the first series of the first session (S1) and the second series of the first session (S2). The dotted lines represent the 95% limits of agreement (*n* = 16).

For the intra- and inter-operator analyses, ICC revealed a high reliability [0.9907–0.9997 (*P* < 0.01) and 0.9989–0.9999 (*P* < 0.01), respectively] for the evaluation of the landing position of the balls. Tables 2, 3 present the intrasession and intersession reproducibility outcomes, respectively. Ball speed, accuracy score and PI meet the criteria of an ICC > 0.70 for reliability. ICC’s were high and significant for intrasession (0.78–0.96, *P* < 0.01) and intersession (0.78–0.94, *P* < 0.05) for all variables. The agreement parameters are also at an acceptable level for all variables, with CV ranged 2.7–16.2% for the intrasession and 6.6–14.5% for the intersession. Moreover, the Bland–Altman plots show the mean difference or systematic error of the intrasession and intersession analyses closer to 0 (ranged -0.5–0.7 and -0.8–0.9, respectively), and that most values were within the limits of agreements (Figure 2, 3 for intrasession and intersession, respectively).

**Table 2:** Intrasession reproducibility outcomes for ball speed, accuracy score, and performance index of the table tennis players. (*n* = 16).

	S1	S2	ICC (*P*-value)	TE	SDD	CV
Ball speed (km/h)	43.1 ± 1.2	43.8 ± 1.1	0.96 (<0.001)	1.2	3.2	2.7
Accuracy score (/66)	21.7 ± 1.4	20.8 ± 1.5	0.82 (0.001)	3.3	9.1	15.0
Performance index	9.3 ± 0.6	9.1 ± 0.6	0.78 (0.004)	1.5	4.1	16.2


*Data are expressed as mean ± SEM. Abbreviations: S1, first series of the first session; S2, second series of the first session; ICC, intraclass coefficient correlation; TE, typical error; SDD, smallest detectable difference; CV, coefficient of variation (%).*

**Table 3:** Intersession reproducibility outcomes for ball speed, accuracy score, and performance index of the table tennis players. (*n* = 12).

	S1	S3	ICC (*P*-value)	TE	SDD	CV
Ball speed (km/h)	42.7 ± 1.5	42.0 ± 1.2	0.78 (0.01)	2.8	7.7	6.6
Accuracy score (/66)	22.8 ± 1.5	23.3 ± 1.9	0.94 (<0.001)	2.1	5.9	9.3
Performance index	9.7 ± 0.6	9.8 ± 0.8	0.86 (0.002)	1.4	3.7	14.5


*Data are expressed as mean ± SEM. Abbreviations: S1, first series of the first session; S3, first series of the second session; ICC, intraclass coefficient correlation; TE, typical error; SDD, smallest detectable difference; CV, coefficient of variation (%).*

**FIGURE 3:**
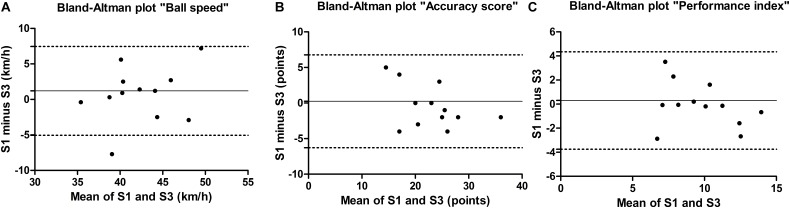
Bland–Altman plots for **(A)** ball speed, **(B)** accuracy score, and **(C)** performance index. The continuous line represents the mean difference between the first series of the first session (S1) and the first series of the second session (S3). The dotted lines represent the 95% limits of agreement (*n* = 12).

The results of discriminant validity analyses are presented in Table 4. Ball speed of RP was slightly higher than LP (+1.7%); however, this difference was not significant (P > 0.05; ES < 0.19). On the other hand, significant higher values of accuracy score (+51.3%) and PI (+56.1%) as well as lower percentage error (-25.4%) were found in RP compared to LP (P < 0.05). ES analysis confirmed a large magnitude of the difference between groups (ES > 0.80) for accuracy score, PI, and percentage errors. More specifically, RP reached the target zones significantly more often than LP (14.5 ± 3.3% for RP and 6.7 ± 1.2% for LP; P = 0.04; ES = 0.95). The right rectangle zone was 7-fold more reached in RP compared to LP (9.4 ± 3.8% for RP and 1.3 ± 0.9% for LP; P = 0.07; ES = 0.90). Percentage of the balls that reached the left rectangle (0.6 ± 0.4% for RP and 1.0 ± 0.7% for LP; P = 0.60; ES = 0.23) and semicircle (4.5 ± 1.0% for RP and 4.4 ± 1.4% for LP; P = 0.92; ES = 0.02) zones were similar between groups.

**Table 4:** Ball speed, accuracy score, performance index for the regional (RP), and local (LP) performance-level groups.

	RP (*N* = 10)	LP (*N* = 9)	*P*-value	Effect Size
Ball speed (km/h)	45.8 ± 1.3	45.0 ± 2.6	0.78	0.13
Accuracy score (/66)	22.7 ± 2.0	15.0 ± 1.7	0.01	1.33
Performance index	10.2 ± 0.6	6.5 ± 0.6	0.0009	1.87
Percentage error (%)	45.5 ± 3.8	60.9 ± 4.6	0.017	1.20


*Data are expressed as mean ± SEM.*

In keeping with the above findings, the association between ball speed and table tennis performance was not significant (r = 0.32, P = 0.49); while, accuracy score (r = -0.79, P = 0.04), PI (r = -0.78, P = 0.04), and percentage error (r = 0.88, P = 0.01) presented a strong and significant correlation with table tennis performance (concurrent validity). This means that players with a high position at the simulated tournament tended to have a higher accuracy score and PI as well as a lower percentage error.

## Discussion

We aimed to develop a table tennis test of stroke effectiveness based on the temporal game structure to assess the ball speed and ball placement of the players, with a purpose to analyze its reproducibility and validity. Our main findings showed the proposed test was reproducible for all variables as well as presented a discriminative and concurrent validity for ball placement and ball velocity-ball placement index, providing promising results for monitoring the stroke effectiveness of the athletes in a tactical situation of offensive strokes performed at defensive balls.

The results for the intra- and inter-operator analyses showed evidence of an almost perfect reliability. Our specific-test was recorded with three cameras, thus the ball trajectory from the contact with the racket to the contact with the table (i.e., landing position) could be accurately registered. Although more complex, this robust methodology is an advantage compared to prior stroke performance test that used a radar system and a visual methodology to evaluate ball speed and placement, respectively (Le Mansec et al., 2016). Furthermore, our results show evidences that our proposed test provides a reproducible instrument for measure ball speed, ball placement, and velocity-accuracy index, which showed high levels of reliability (ICC range 0.78–0.96) and satisfactory levels of agreement (TE range 1.2–2.8 and CV range 2.7–16.2%) when players performed the test in a short period or several days later. These corroborate with the ICC ranging from 0.45 to 0.96 and CV ranging from 2 to 19% presented in reproducibility studies on the assessment of technical performance (Le Mansec et al., 2016), tactical-technical performance (Vergauwen et al., 1998; Kolman et al., 2017), perceptuo-motor skills (Faber et al., 2014a, 2015), and cognitive performance (van de Water et al., 2017) in racket sports players. Moreover, Bland–Altman plots showed the mean difference of the intrasession and intersession analyses closer to zero in our study, which represent reliable measures.

Concerning discriminant validity, we showed regional players had higher scores of ball placement and ball velocity-ball placement index as well as made fewer errors than local players. Prior studies applied to racket sport concur reporting the scores of ball placement, ball velocity-ball placement index, and percentage error discriminate players with different performance-level during technical (Le Mansec et al., 2016) and tactical-technical tests (Vergauwen et al., 1998; Kolman et al., 2017). Lateral stroke precision also tended to be better in RP than LP. Corroborating this result, Vergauwen et al. (1998) showed evidence the lateral stroke precision was relevant to discriminate tennis players with different levels of performance in a technical-tactical test. Moreover, Djokic (2002) observed that better ranked table tennis players (by the position on the International Table Tennis Federation Rank List) more use the lateral stroke placement compared to lower ranked players during official matches. Thus, further research should better explore the accuracy analysis of the lateral target areas to continue the development of table tennis tests. For example, the right zone was more reached than the left zone for regional players (15.5-fold on average). It is plausible to assume that players preferred to better explore the right than the left area, given the athletes could hit the targets in a sequence they decided. Splitting right area in half, we may also visualize the regional players reached most balls in half of the target closer to the edge (71%) than in half of the target closer to the net (29%). Strokes aimed toward the end part of the target stimulated higher ball speed (∼13%) while hitting the target closer to the net provided bigger angle, which are different ways to move the ball out of the opponent control during a real match. Finally, further research may also analyze which stroke was more effective to reach these areas, given the athletes could choose among different FH and BH techniques for offensive strokes to perform the proposed test [e.g., initial topspin, fast final topspin attack, preparatory drive attack, and final drive attack (Munivrana et al., 2015b)].

Ball speed slight differed between the groups during the proposed test. We focused on the successful shots to measure the ball speed, which could influence our results and constitute a possible explanation concerning the slight difference found between the groups. However, Vergauwen et al. (1998) also measure the ball speed in nonerror strokes and they observed the ball speed was relevant to discriminate tennis players with different performance-level during a test to examine stroke effectiveness in match-like conditions. An alternative explanation is table tennis players can use different playing styles. Offensive players tend to win the point by accelerating the speed of play (Martin et al., 2015) whereas all-round players prefer to return the ball and to force opponent’s error, with less aggressive strokes (Milioni et al., 2018). The study penholder players used an offensive playing style while players who used classical racket grip were all-round players. In line with this, we found a lower percentage of offensive players in regional (10%) than local (33%) groups, which could partially clarify our findings. Finally, the ball speed and ball spin are often related to and restricted by each other (Qun et al., 1992). Thus, it is possible that regional players may have increased the use of ball spin during their strokes to increase the accuracy, but it reduced the velocity they hit the ball to perform the test. This is in accordance with the speed-accuracy trade-off (i.e., to reach greater accuracy, the execution time of a movement increases) (Fitts, 1954). Considering this assumption, it would be interesting to further investigate whether higher performance-level players are able to be accurate in their strokes with a lower impact on the ball speed than regional players during our test.

Concurrent validity concur the aforementioned findings, showing that the ball placement, PI, and percentage error were related with table tennis performance whereas ball speed was not associated with the players’ position on the simulated tournament. Le Mansec et al. (2016) reported the scores of ball placement and ball velocity-ball placement index were related with the table tennis performance; however, this investigation also observed the ball speed evaluated during a continuous and specific-test was related with the players’ position on the national ranking list.

Herein, the test explores the phase of attack with offensive strokes at defensive balls delivered by a robot close to the edge of the table, which could constitute a limitation of this investigation. Besides elements used in the phase of attack, the whole structure of technical-tactical elements in table tennis also includes those used in the phase of defense, and those used in the phase of preparing one’s own and disabling the opponent’s attack, which are performed at balls bounce in different playing surfaces over the table (i.e., net zone, middle zone, baseline/edge zone) (Munivrana et al., 2015a,b). In line with this, development of technical-tactical tests applied to tennis proposed different tactical situations (i.e., offensive, neutral and defensive rallies) by varying the direction of the ball sent by robot (Vergauwen et al., 1998; Kolman et al., 2017). In addition, changes in frequency of balls delivered by the robot may also be explored during different rallies (e.g., decreases the time between strokes could be informative as time pressure in defensive rallies). These issues may be explored in future investigations to continue the development of table tennis tests based on the temporal game structure started at present investigation. Another limitation is related to the table tennis performance analyses. We evaluated three different teams, each one ranked from a different table tennis circuit; thus, a simulated tournament constituted an alternative to rank the study players according their performance during real matches. However, the use of the simulated tournament as a measure of performance is a point of discussion, because the players’ performance is based on only one moment of competition without a broader performance context as those observed in a table tennis circuit. Thus, it is interesting for future research to investigate whether the test is related to the table tennis players’ position on a ranking list based on a table tennis circuit. Finally, future studies may also focus on designing tests to evaluate national and international players, since it is documented that the temporal game structure of table tennis is influenced by the performance level (Zagatto et al., 2010; Leite et al., 2017) and the results observed herein are representative of regional players.

## Conclusion

This study presents a novel table tennis test of stroke effectiveness based on the temporal game structure, which is able to assess the ball speed, ball placement and the interrelation between these two tactical means in the players. This is the first study which the proposed specific-test were based on temporal characteristics of the table tennis match. Our findings show evidences that the test is reproducible, with satisfactory reliability and agreement outcomes. Moreover, discriminant and concurrent validity are confirmed, except for individual scores on ball speed. Practical implications for coaches can be to use this test at the beginning and at the end of a teaching and training period, for monitoring the effectiveness of offensive strokes of the players to deal with defensive opponents. Differences between the second and the first test session on the PI larger than the SDD of 3.7–4.1 are indicative of athletes’ improvement within a teaching and training period.

## Data Availability

The datasets generated for this study are available on request to the corresponding author.

## Author Contributions

TB and PdM designed the study, conducted analyses, and wrote the manuscript. MM, TT, RR, YS, and KS assisted in acquisition, analysis and interpretation of data, and reviewed the article. LG made substantial contribution including conception of the study and a critical revision of the article. All the authors read and approved the final manuscript.

## Conflict of Interest Statement

The authors declare that the research was conducted in the absence of any commercial or financial relationships that could be construed as a potential conflict of interest.
